# Ubiquitous lognormal distribution of neuron densities in mammalian cerebral cortex

**DOI:** 10.1093/cercor/bhad160

**Published:** 2023-07-06

**Authors:** Aitor Morales-Gregorio, Alexander van Meegen, Sacha J van Albada

**Affiliations:** Institute of Neuroscience and Medicine (INM-6) and Institute for Advanced Simulation (IAS-6) and JARA-Institut Brain Structure-Function Relationships (INM-10), Jülich Research Centre, Wilhelm-Johnen-Str., 52428 Jülich, Germany; Institute of Zoology, University of Cologne, Zülpicher Str., 50674 Cologne, Germany; Institute of Neuroscience and Medicine (INM-6) and Institute for Advanced Simulation (IAS-6) and JARA-Institut Brain Structure-Function Relationships (INM-10), Jülich Research Centre, Wilhelm-Johnen-Str., 52428 Jülich, Germany; Institute of Zoology, University of Cologne, Zülpicher Str., 50674 Cologne, Germany; Institute of Neuroscience and Medicine (INM-6) and Institute for Advanced Simulation (IAS-6) and JARA-Institut Brain Structure-Function Relationships (INM-10), Jülich Research Centre, Wilhelm-Johnen-Str., 52428 Jülich, Germany; Institute of Zoology, University of Cologne, Zülpicher Str., 50674 Cologne, Germany

**Keywords:** neuron density, cytoarchitecture, lognormal distribution, neurogenesis, mathematical model

## Abstract

Numbers of neurons and their spatial variation are fundamental organizational features of the brain. Despite the large corpus of cytoarchitectonic data available in the literature, the statistical distributions of neuron densities within and across brain areas remain largely uncharacterized. Here, we show that neuron densities are compatible with a lognormal distribution across cortical areas in several mammalian species, and find that this also holds true within cortical areas. A minimal model of noisy cell division, in combination with distributed proliferation times, can account for the coexistence of lognormal distributions within and across cortical areas. Our findings uncover a new organizational principle of cortical cytoarchitecture: the ubiquitous lognormal distribution of neuron densities, which adds to a long list of lognormal variables in the brain.

## Introduction

Neurons are not uniformly distributed across the cerebral cortex; their density varies strongly across areas and layers (Brodmann 1909; Economo et al. 2008). The neuron density directly affects short-range as well as long-range neuronal connectivity (Braitenberg and Schüz 1991; Ercsey-Ravasz et al. 2013). Elucidating the distribution of neuron densities across the brain therefore provides insight into its connectivity structure and, ultimately, cognitive function. Additionally, statistical distributions are essential for the construction of computational network models, which rely on predictive relationships and organizational principles where the experimental data are missing (Hilgetag et al. 2019; Albada et al. 2022). Previous quantitative studies have provided reliable estimates for cell densities across the cerebral cortex of rodents (Herculano-Houzel et al. 2013; Charvet et al. 2015; Erö et al. 2018), non-human primates (Collins et al. 2010; Charvet et al. 2015; Collins et al. 2016; Turner et al. 2016; Atapour et al. 2019; Beul and Hilgetag 2019), large carnivores (Jardim-Messeder et al. 2017), and humans (Economo et al. 2008; Bartheld et al. 2016). However, to the best of our knowledge, the univariate distribution of neuron densities across and within cortical areas has not yet been statistically characterized. Instead, most studies focus on qualitative and quantitative comparisons across species, areas, or cortical layers. Capturing the entire distribution is necessary because long-tailed, highly skewed distributions are prevalent in the brain (Buzsáki and Mizuseki 2014) and invalidate the intuition—guided by the central limit theorem—that the vast majority of values are in a small region of a few standard deviations around the mean.

**Fig. 1:**
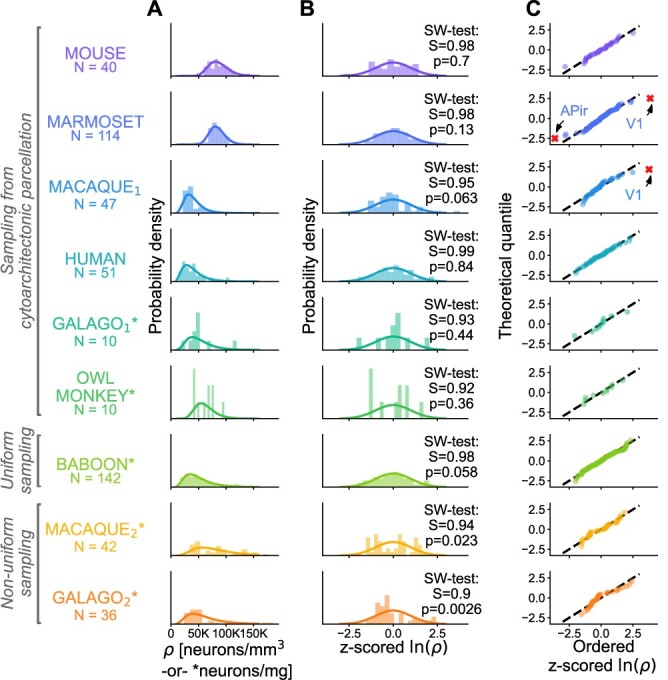
Neuron and cell densities $\rho $ follow a lognormal distribution across cortical areas for multiple species. Different data sets for the same species are denoted with subscript indices (see Section Cell density data). (A) Histogram of $\rho $ (bars) and probability density function of a fitted lognormal distribution (line). The number of samples $N$ is either the number of sampled cytoarchitectonic areas (mouse, marmoset, macaque$_1$, human, galago$_1$, and owl monkey) or the number of sampling frames (baboon, macaque$_1$, and galago$_2$). For marmoset, galago, owl monkey, baboon, and macaque$_2$, the data are based on a single subject; for mouse, macaque$_1$, and human it is based on a combination of data across several subjects. (B) Z-scored $\ln (\rho )$ histogram (bars), standard normal distribution (line), and result of the SW normality test. (C) Probability plot of z-scored $\ln (\rho )$. Discarded outliers marked with a red cross.

Here, we characterize the distribution of neuron densities $\rho $ across mammalian cerebral cortex. Based on the sample histograms (Fig. 1) we hypothesize that $\rho $ follows a lognormal distribution, similar to many other neuroanatomical and physiological variables (Buzsáki and Mizuseki 2014) such as synaptic strengths (Robinson et al. 2021), synapse sizes (Loewenstein et al. 2011; Santuy et al. 2018; Dorkenwald et al. 2022), axonal widths (Wang et al. 2008; Liewald et al. 2014), and cortico-cortical connection densities (Markov et al. 2014; Gămănuţ et al. 2018). We used neuron density data from mouse (*Mus musculus*), marmoset (*Callithrix jacchus*), macaque (*Macaca mulatta*), human (*Homo sapiens*), galago (*Otolemur garnettii*), owl monkey (*Aotus nancymaae*), and baboon (*Papio cynocephalus anubis*) to test this hypothesis (see Section Cell density data for a detailed description of the data). The marmoset, galago, owl monkey, baboon, and macaque$_2$ data sets are based on a single subject; the mouse, macaque$_1$, and human data sets are based on a combination of data across several subjects. The statistical tests conclude that the hypothesis cannot be rejected in the majority of cases, suggesting that the underlying distribution is compatible with a lognormal distribution if the samples are based either on cytoarchitectonically defined areas or on uniformly sampled regions. Beyond the distribution across cortical areas, we show that neuron densities within most areas of marmoset cortex are also compatible with a lognormal distribution. To complement the statistical tests, we perform a model comparison with several other distributions and find that none outperform the lognormal distribution as a model of the data within and across areas. Finally, we show that the lognormal distribution within cortical areas can emerge during neurogenesis from a simple cell division model with variability. The model can furthermore account quantitatively for the lognormal density distribution across areas based on an inferred distribution of proliferation times of the areas. Additional between-area variability in the proliferation rates is also compatible with the model but not necessary to obtain a quantitative agreement with the observed distribution. Thus, our model shows how the lognormal distribution of neurons could emerge both within and across the cortical areas.

## Materials and methods

### Cell density data

Estimates of neuron density for the available cortical areas across the mouse (*Mus musculus*), marmoset (*Callithrix jacchus*), macaque (*Macaca mulatta*), human (*Homo sapiens*), galago (*Otolemur garnettii*), owl monkey (*Aotus nancymaae*), and baboon (*Papio cynocephalus anubis*) cerebral cortex were used in this study.

In the cases of mouse, marmoset, macaque$_1$, human, galago$_1$, and owl monkey the data were sampled from standard cytoarchitectonic parcellations; abbreviated names for all areas are listed in Table S1. Note that we use subscript indices to distinguish between different data sets on the same model animal, e.g. macaque$_1$ and macaque$_2$.

Neuron density estimates for the mouse were published by Erö et al. (2018), and were measured from previously published Nissl-body-stained slices (Lein et al. 2007), where genetic markers were used to distinguish between cell types. The data were provided in the Allen Brain Atlas parcellation (Lein et al. 2007; Dong 2008). To estimate the quantitative neuron and cell densities Erö et al. (2018) used “a variety of whole brain image datasets [from the Allen Mouse Brain Atlas]” as well as “some values reported from anatomical experiments in the literature”, such as from (Herculano-Houzel et al. 2013).

Neuron density estimates for the marmoset cortex were published by Atapour et al. (2019), and were measured from NeuN-stained slices. The data were provided in the Paxinos parcellation (Paxinos et al. 2011) and all quantitative values are derived from the same brain of a single subject. Neuron densities within each counting frame used in the original publication (Atapour et al. 2019, their Fig. S1) were obtained via personal communication with Nafiseh Atapour, Piotr Majka, and Marcello G. Rosa.

The neuron density estimates in the first macaque data set, macaque$_1$, were previously published in visual form by Beul and Hilgetag (2019), and were obtained from both Nissl-body- and NeuN-stained brain slices. Most of the numerical values were reported by Dombrowski et al. (2001) and Hilgetag et al. (2016), and the remaining values were provided by Sarah F. Beul and Claus C. Hilgetag via personal communication. Counts based on Nissl-body staining were scaled according to a linear relationship with the counts from NeuN staining obtained from selected areas where both types of data were available (Beul and Hilgetag 2019). The data follow the M132 parcellation (Markov et al. 2014) and constitute the average across subjects.

Cell density estimates for the human cortex were previously published by Economo et al. (2008), and were measured from Nissl-body-stained brain slices. The human data therefore most likely reflect combined neuron and glia densities. The data were provided in the von Economo parcellation (Economo et al. 2008); all quantitative values are based on “the mean of the numbers gathered from the various brains” (Economo et al. 2008, p. 201].

Cell and neuron density estimates for galago$_{1\&2}$, owl monkey, baboon, and macaque$_2$ were previously published by Collins et al. (2010), and were measured using the isotropic fractionator method. The data are sampled from common parcellation schemes in galago$_1$ and owl monkey, approximately equal-size samples in the baboon, and non-uniform samples in macaque$_2$ and galago$_2$. We refer to uniform sampling when the cortex was subdivided into small regions of approximately equal size and shape presumably without large cytoarchitectonic variations (the case for the baboon), and to non-uniform sampling when the samples often cross cytoarchitectonic boundaries (the case for macaque$_2$ and galago$_2$). All the quantitative values were derived from one hemisphere of each subject separately. Each data set thus describes only a single subject, without combining data across individuals of the same species.

Note that all the samples representing either full cortical areas or crossing area boundaries include the entirety of the gray matter, spanning all layers of cortex. The only layer-resolved data set is the marmoset; here the mean across all layers was reported as the density.

### Lognormality testing

To test for lognormality, we take the natural logarithm, $\ln (\rho )$, which converts lognormally distributed samples into normally distributed samples. We then test for normality of $\ln (\rho )$ using the Shapiro-Wilk (SW) test, the most powerful among a number of commonly applied normality tests (Razali and Yap 2011). Large outliers ($\rm |$z-scored $\ln (\rho ) \rm | \geq 3$) were excluded from the normality test; the excluded outliers are indeed cytoarchitectonically distinct areas (discussed below).

Note that any hypothesis test, including the SW test, cannot show that the distribution is lognormal. If the *p*-value is larger than a certain threshold one cannot reject the null hypothesis that the distribution of $\rho $ is lognormal, i.e. the data are compatible with a lognormal distribution. Thus, we perform further tests, such as statistical model comparison, and comparing $\ln (\rho )$ across different animals with a Kolmogorov-Smirnov test.

### Statistical model comparison

In order to assess which model is most compatible with the data, we compared the relative likelihood of different distributions against each other. We included an extensive list of distributions with support on $\mathbb{R}^+$, estimated the distributions’ parameters using maximum likelihood, and calculated the Akaike Information Criterion ($AIC$) 


(1) \begin{align*}& AIC = 2k - 2\ln{\mathcal{L}} \end{align*}

where $k$ is the number of estimated parameters of the model and $\mathcal{L}$ is the estimated maximum likelihood. We further compare the models using the relative likelihood ($\mathcal{L}_r$) 


(2) \begin{align*}& \mathcal{L}_r = e^{(AIC_{\text{min}} - AIC_i)/2} \end{align*}

where $AIC_{\text{min}}$ is the minimum $AIC$ across all models and $AIC_i$ is the $AIC$ for the $i$th model. The relative likelihood indicates the probability that, from among the tested models, the $i$th model most strongly limits the information loss (Burnham and Anderson 2004). We take a significance threshold of $\alpha =0.05$ on the relative likelihood to determine whether a model is significantly worse than the best possible model.

### Model of neurogenesis with variability

#### Within areas

Neurons are generated through symmetric or asymmetric cell division of neural progenitor cells (Cadwell et al. 2019). Grouping all types of progenitor cells into a single population $P$, all neurons into a population $N$, and excluding other post-mitotic cell types, their population size can be modeled by the coupled system of differential equations (Picco et al. 2018) 


(3) \begin{align*}& \frac{d}{dt}P=\lambda_{P}P-\lambda_{N}P,\qquad\frac{d}{dt}N=\lambda_{N}P, \end{align*}

where $\lambda _P$ denotes the (potentially time-dependent) rate of progenitor generation and $\lambda _N$ the (potentially time-dependent) rate of neuron generation.

Following the radial unit hypothesis (Rakic 2009), we consider a small number of such radial units (small compared to the total size of the area) and determine the density of progenitors $\rho _P$ and neurons $\rho _N$ in these radial units by dividing through the volume $V$ of the considered radial units in the fully developed cortex. Importantly, this reference volume is the same for every area. Since Equation (3) is linear, dividing by $V$ leads to 


(4) \begin{align*}& \frac{d}{dt}\rho_P=\lambda_{P}\rho_P-\lambda_{N}\rho_P,\qquad\frac{d}{dt}\rho_N=\lambda_{N}\rho_P. \end{align*}

Note that this does not necessarily exclude tangential migration as long as the net influence of the tangential migration is zero.

**Progenitor cell proliferation** First, we consider the proliferation of the progenitor cells, which we assume to be governed by a noisy rate. Modeling the noise by a zero-mean, unit-strength Gaussian white noise process $\xi $, we obtain a stochastic differential equation (SDE) for the progenitor cell density 


(5) \begin{align*}& \frac{d}{dt}\rho_{P}=(\lambda_{P}-\lambda_{N}+\sigma\xi)\rho_{P} \end{align*}

where $\sigma $ controls the (potentially time-dependent) intensity of the noise. Using the Stratonovich interpretation—assuming that the noise process has a small but finite correlation time before taking the white-noise limit (Van Kampen 2007)—the SDE transforms by the same rules as an ordinary differential equation (Van Kampen 2007) such that we can rewrite the SDE (5) as $\frac{d}{dt}\ln \rho _{P}=\lambda _{P}-\lambda _{N}+\sigma \xi $ with the solution 


(6) \begin{align*}& \ln\rho_{P}(t)=\ln\rho_{0}+\int_0^t \big(\lambda_{P}(s)-\lambda_{N}(s)\big)ds+\int_{0}^{t}\sigma(s)\xi(s)ds. \end{align*}

Since $\xi (t)$ is Gaussian and Equation (6) is linear, $\ln \rho _{P}(t)$ is Gaussian and hence $\rho _{P}(t)$ is lognormally distributed at all times $t$. The parameters of this lognormal distribution are the mean of the logarithmic progenitor cell density $\mu _{P}(t)=\langle \ln \rho _{P}(t)\rangle $ and the variance of the logarithmic progenitor cell density $\sigma _{P}(t)^{2}=\langle \Delta (\ln \rho _{P}(t))^{2}\rangle $ (here $\Delta x \equiv x - \langle x \rangle $). Using Equation (6), $\langle \xi (s)\rangle =0$, and $\langle \xi (s)\xi (s^{\prime })\rangle =\delta (s-s^{\prime })$, we obtain (cf. for instance Braumann (2007)) 


(7) \begin{align*}& \begin{aligned} \mu_{P}(t)&=\ln\rho_{0}+\int_0^t \big(\lambda_{P}(s)-\lambda_{N}(s)\big)ds, \\ \sigma_{P}(t)^{2}&=\int_{0}^{t}\sigma(s)^{2}ds. \end{aligned} \end{align*}

Thus, the progenitor cell densities are lognormally distributed at all times with parameters $\mu _{P}(t)$ and $\sigma _{P}(t)^2$. The corresponding first 2 moments of the progenitor cell density are 


(8) \begin{align*}& \begin{aligned} \langle \rho_{P}(t)\rangle &=e^{{\mu_{P}(t)+\frac{1}{2}\sigma_{P}(t)^{2}}}, \\ \langle \rho_{P}(t)\rho_{P}(t^{\prime})\rangle&=\langle \rho_{P}(t)\rangle\langle \rho_{P}(t^{\prime})\rangle e^{{\int_{0}^{\min(t,t^{\prime})}\sigma(s)^{2}ds}}, \end{aligned} \end{align*}

where we used the characteristic functional of the Gaussian white noise $\xi (t)$ (Van Kampen 2007), $\langle \exp (i\int _{-\infty }^{\infty } k(s)\sigma (s)\xi (s)ds) \rangle = \exp (-\frac{1}{2} \int _{-\infty }^{\infty } \sigma (s)^2k(s)^2)$, with the test function $ik(s)=1\!\!1_{[0,t]}(s)$ for the first moment (here $1\!\!1_A(s)$ denotes the indicator function) and test function $ik(s)=1\!\!1_{[0,t]}(s)+1\!\!1_{[0,t^\prime ]}(s)$ for the second moment as well as $\int _{-\infty }^{\infty } 1\!\!1_{[0,t]}(s)1\!\!1_{[0,t^\prime ]}(s)\sigma (s)^2 ds = \int _{0}^{\min (t,t^{\prime })} \sigma (s)^2 ds$.

**Neurogenesis** Next, we consider neurogenesis. We assume that the noise affects primarily the rate of progenitor cell proliferation. The solution to Equation (4) for a given $\rho _{P}(t)$ and initial condition $\rho _{N}(0)=0$ is 


(9) \begin{align*}& \rho_{N}(t)=\int_{0}^{t}\lambda_{N}(s)\rho_{P}(s)ds. \end{align*}

Since $\rho _{N}(t)$ is the integral of the (temporally correlated) lognormal process $\rho _{P}(s)$ it is formally not lognormal. However, the sum of independent lognormal random variables is well approximated by a lognormal distribution with matched first and second moment (Fenton 1960; Marlow 1967). Here, we extend this approximation to the integral of temporally correlated lognormal processes. The first 2 moments of the neuron density follow from the averages of Equation (9), 


(10) \begin{align*}& \begin{aligned} \langle \rho_{N}(t)\rangle&=\int_{0}^{t}\lambda_{N}(s)\langle \rho_{P}(s)\rangle ds,\\ \langle \rho_{N}(t)^{2}\rangle&=\int_{0}^{t}\int_{0}^{t}\lambda_{N}(s)\lambda_{N}(s^{\prime})\langle \rho_{P}(s)\rho_{P}(s^{\prime})\rangle dsds^{\prime}. \end{aligned} \end{align*}

The lognormal approximation with matched moments is parameterized by 


(11) \begin{align*}& \begin{aligned} \mu_{N}(t)&=\ln\langle \rho_{N}(t)\rangle-\frac{1}{2}\sigma_{N}(t)^{2}, \\ \sigma_{N}(t)^{2}&=\ln\Bigg(1 + \frac{\langle \Delta(\rho_{N}(t))^{2}\rangle}{\langle \rho_{N}(t)\rangle^{2}}\Bigg), \end{aligned} \end{align*}

where we used $\langle x \rangle = e^{\mu + \tfrac{1}{2}\sigma ^2}$ and $\langle \Delta x^2 \rangle = \langle x \rangle ^2 [e^{\sigma ^2}-1]$ for a lognormal variable $x$. Note that the parameters of the lognormal distribution are the mean of the logarithmic neuron density $\mu _{N}(t)=\langle \ln \rho _{N}(t)\rangle $ and the variance of the logarithmic neuron density $\sigma _{N}(t)^{2}=\langle \Delta (\ln \rho _{N}(t))^{2}\rangle $.

#### Across areas

Thus far, the model accounts for the lognormal distribution of neuron densities within an area. Across areas, we hypothesize that the distribution of proliferation times (Rakic 2002; Cadwell et al. 2019) is the most important cause of the variability in the neuron densities. To characterize an individual area, we consider the average density $\langle \rho _N(t)\rangle $. For convenience, we introduce the auxiliary quantity 


(12) \begin{align*}& \begin{aligned} y(t)=\ln\bigg(\int_{0}^{t}\lambda_{N}(s)e^{\int_{0}^{s}(\lambda_{P}(r)-\lambda_{N}(r))dr+\frac{1}{2}\int_{0}^{s}\sigma(r)^{2}dr}ds\bigg) \end{aligned} \end{align*}

such that the logarithm of the mean neuron density simplifies to 


(13) \begin{align*}& \ln\langle\rho_{N}(t)\rangle = \ln\rho_{0} + y(t) \end{align*}

where we inserted Equation (7) into Equation (8) to obtain the mean of the progenitor cell density, which we then plugged into Equation (10).

In order to arrive at a lognormal distribution of the mean density $\langle \rho _{N}(t)\rangle $ across areas, the terms on the r.h.s. of Equation (13) have to be normally distributed. In particular, this means that the proliferation times have to be distributed such that $y(t)$ is Gaussian. The distribution $p(y)$ of $y=y(t)$, which is a strictly monotonic transformation of the proliferation time $t$, is related to the distribution of proliferation times $p(t)$ through (Van Kampen 2007) 


(14) \begin{align*}& p(t) = \Big|\frac{dy}{dt}\Big|\,\mathcal{N}\big(y(t)\,|\,\mu_{y},\sigma_{y}^{2}\big). \end{align*}

The first factor on the r.h.s. can be obtained from Equation (12) and the second factor is a Gaussian probability density with mean $\mu _y$ and variance $\sigma _y^2$. Hence, Equation (14) fully specifies the distribution of proliferation times; conversely, for proliferation times distributed according to Equation (14), the mean neuron density $\langle \rho _{N}(t)\rangle $ is lognormally distributed across areas. Note that since the Gaussian has support on the entire real line, the neuron density needs to diverge for $t\to \infty $. Thus, we restrict $\lambda _{P}(t)$, $\lambda _{N}(t)$, and $\sigma (t)$ such that the neuron density would diverge in the hypothetical limit of an infinite proliferation time.

#### Parameter estimation

Above, we showed how a noisy rate of progenitor proliferation leads to a lognormal distribution of progenitor cell densities and neuron densities within an area and, for the distribution of proliferation times (14), to a lognormal distribution of (within-area) mean neuron densities across areas. In order to compare the predictions of the model with the data, we estimate the model’s parameters using the available experimental data.

We restrict the analysis to the simplified case of constant rate $\lambda _{P}$, $\lambda _{N}$, and noise intensity $\sigma $ and identical rates of progenitor and neuron proliferation, $\lambda _{P}=\lambda _{N}\equiv \lambda $. In particular the assumption of a constant rate (note that this is not a necessary assumption for the above theory) is simplifying because the cell cycle length varies during development (Kornack and Rakic 1998). Despite this simplifying assumption, the model quantitatively matches the data for the parameters inferred below (Fig. 5).

First, we determine the rate $\lambda $ from the average cell cycle length of progenitor cells determined from a short period of $2$ hours (Kornack and Rakic 1998) such that fluctuations and the conversion of progenitor cells to neurons can be neglected. For a given cell cycle length $\ell $, the number of cells increases as $2^{t/\ell }=\exp \big (t\ln (2)/\ell \big )$. Thus, the cell cycle length $\ell $ corresponds to a proliferation rate $\lambda =\ln (2)/\ell $. Using the average cell cycle length of $\ell \approx 1.5\,\text{days}$ from macaque (Kornack and Rakic 1998; Picco et al. 2018), we obtain $\lambda \approx 0.46\,\text{days}^{-1}$. The proliferation time of areas varies between 30 and 60 days in macaque (Rakic 2002) which we expect to be similar in the marmoset since macaques and marmosets have similar gestation times of 5.5 and 4.5 months, respectively (Schultz-Darken et al. 2016). Thus, we set the median proliferation time per area to $t_{1/2}=45$ days, which determines $\mu _y=y(t_{1/2})$ because the median of the distribution (14) is given by $\frac{1}{2}=\int _0^{t_{1/2}} p(t) dt = \int _{-\infty }^{y(t_{1/2})} \mathcal{N}(y\,|\,\mu _y, \sigma _y^2) dy$ and the median of the normal distribution is at $y=\mu _y$.

In addition, the mean $\mu _y$ of the auxiliary variable $y$ is also constrained by the distribution of the variance $\sigma _{N}^{2}$ of the logarithmic neuron density across areas; we will use this additional constraint to determine the noise intensity $\sigma $. For a fixed proliferation time $t$, $\sigma _{N}(t)^{2}$ is given by Equation (11). Since $\sigma _{N}^{2}$ is a strictly monotonically increasing function of $t$, its distribution can be written in terms of that of the inverse $t(\sigma _{N}^{2})$, i.e. $p(\sigma _{N}^{2})=\Big |\frac{dt}{d\sigma _{N}^{2}}\Big |\,p\big (t(\sigma _{N}^{2})\big )$. Using Equation (14) for the distribution of proliferation times $p(t)$ and Equation (12) to relate $y(t)$ and $t$, the distribution of $\sigma _{N}^{2}$ across areas is thus 


(15) \begin{align*}& p(\sigma_{N}^{2})=\frac{1}{|f^{\prime}(f^{-1}(\sigma_{N}^{2}))|}\,\mathcal{N}\big(f^{-1}(\sigma_{N}^{2})\,|\,\mu_{y},\sigma_{y}^{2}\big) \end{align*}

where $f(y)=\sigma _{N}^{2}(t(y))$ with $t(y)$ the inverse of 


(16) \begin{align*}& y(t)=\ln\frac{2\lambda}{\sigma^2}+\ln(e^{\frac{1}{2}\sigma^2t}-1), \end{align*}

which follows from evaluating Equation (12) with $\lambda _{P}=\lambda _{N}\equiv \lambda $ and constant $\sigma ^2$, and $f^{-1}(\sigma _{N}^{2})$ is the inverse of $f(y)$. Explicitly, it is given by 


(17) \begin{align*}& f^{-1}(\sigma_{N}^{2})=\ln\frac{\lambda}{\sigma^{2}}+\ln\Big(\sqrt{8\big(3e^{\sigma_{N}^{2}}-1\big)}-4\Big), \end{align*}

which follows from inserting $t(y)$ into $\sigma _{N}^{2}(t)$ determined by Equation (11) and solving it for $y$. The maximum likelihood estimator $\hat{\mu }_y$ for $\mu _y$ with the likelihood $p(\sigma _{N}^{2})$ is the empirical average of $f^{-1}(\sigma _{N}^{2})$ across areas because the Jacobian $1/|f^{\prime }(f^{-1}(\sigma _{N}^{2}))|$ in Equation (15) does not depend on the parameters and hence does not affect the Gaussian likelihood of $\mu _y$. Using the marmoset data, we obtain $\hat{\mu }_y\approx 3.07$. We finally arrive at $\sigma \approx 0.061\,\text{days}^{-1/2}$ by enforcing $\hat{\mu }_y=y(t_{1/2})$, where $y(t)$ is given by Equation (16), with $t_{1/2}=45$ days. With $\sigma $ and $\mu _y$ determined, we choose $\sigma _y$ such that less than $2$% of the proliferation times are smaller than 30 days or larger than 60 days. This leads to $\sigma _y^2 \approx 0.02$.

It remains to estimate the initial progenitor density $\rho _0$. To this end, we consider the distribution of $\ln \langle \rho _{N}\rangle $. For a fixed proliferation time $t$, $\ln \langle \rho _{N}(t)\rangle $ is determined by Equation (13). Thus, it is also a monotonic transformation of the random variable $t$, with distribution $p(\ln \langle \rho _{N}\rangle )=\Big |\frac{dt}{d\ln \langle \rho _{N}\rangle }\Big |\,p\big (t(\ln \langle \rho _{N}\rangle )\big )$. Using the linear dependence between $\ln \langle \rho _{N}\rangle $ and $y$, Equation (13), in combination with the distribution of proliferation times (14), we obtain 


(18) \begin{align*}& p(\ln\langle\rho_{N}\rangle)=\mathcal{N}\big(\ln\langle\rho_{N}\rangle\,|\,\ln\rho_{0}+\mu_{y},\sigma_{y}^{2}\big). \end{align*}

The maximum likelihood estimator for $\ln \rho _{0}+\mu _{y}$ is the empirical average of $\ln \langle \rho _{N}\rangle $ across areas; subtracting $\mu _{y}$ we obtain $\rho _{0}\approx 3.8\times 10^3\,\text{cells}/\text{mm}^3$.

Note that in the simplified case considered here, the resulting distribution of proliferation times approaches a lognormal distribution in the left tail and a Gaussian in the right tail: for small times, $\frac{1}{2}\sigma ^2t\ll 1$, a Taylor expansion of $y(t)$ leads to $y(t)\approx \ln (\lambda t)$ and thus a lognormal distribution; for large times, $\frac{1}{2}\sigma ^2t\gg 1$, $y(t)$ grows linearly with $y(t)\approx \ln \frac{2\lambda }{\sigma ^2}+\frac{1}{2}\sigma ^2t$ and thus the distribution approaches a Gaussian.

**Simulation details** We solve the SDE (5) using the Euler-Maruyama method with time step $\Delta t = 0.05$ in total $N_{\text{sample}}$ times with identical parameters for each of the $114$ areas. Because the number of samples per area $N_{\text{sample}}$ varies, we randomly choose $N_{\text{sample}}$ for each area following $N_{\text{sample}} \sim \text{Poisson}(36.6)$ where $36.6$ is the average sample size per cortical area in the marmoset data. The subsequent analysis of the model data is identical to the analysis of the experimental data.

## Results

### Lognormal distribution of neurons across cortical areas

We consider the neuron density distribution across cortex for several species (see Section Cell density data). The SW test (see Section Lognormality testing) concludes that the normality hypothesis of $\ln (\rho )$ cannot be rejected for mouse, marmoset, macaque$_1$, human, galago$_1$, owl monkey, and baboon (Fig. 1B). For the data sets macaque$_2$ and galago$_2,$ the normality hypothesis is rejected (*P*$<0.05$); however, in these data sets, the densities were sampled neither uniformly nor based on a cytoarchitectonic parcellation. The normality hypothesis for the distribution of logarithmic densities across cytoarchitectonic areas is further supported by Figure 1C, which shows that the relation between theoretical quantiles and ordered samples is almost perfectly linear except for macaque$_2$ and galago$_2$.

For lognormality testing, we removed the large outliers (marked with a red cross in Fig. 1C). The outliers are area $\rm V1$ in macaque$_1$ and marmoset, which have densities far outside the range for all other areas in both species, and area $\rm APir$ in marmoset, which has a noticeably distinct cytoarchitecture with respect to the rest of the cerebral cortex (Atapour et al. 2019).

Next, we test the z-scored $\ln (\rho )$ from the different species and data sets against each other and find that they are pairwise statistically indistinguishable ($\alpha = 0.05$ level; two-sample two-sided Kolmogorov-Smirnov test, see Fig. S1 for full test results).

Additionally, we control for cell types in the distributions of the mouse, galago$_1$, owl monkey, and baboon data. In the mouse data, different types of neurons and glia were labeled with specific genetic markers and their respective densities were reported separately for all cell types (Erö et al. 2018). In the galago$_1$, owl monkey, and baboon data sets, the total numbers of cells and neurons were reported separately (Collins et al. 2010). We show that the neuron density distributions for all subtypes of neurons in the mouse are compatible with a lognormal distribution (Fig. S2; SW test on $\ln (\rho )$, $P>0.05$) while glia are not—with the notable exception of oligodendrocytes. When neurons and glia are pooled together (Fig. S2C and D), the distribution of $\ln (\rho )$ still passes the SW normality test, likely due to the distribution being dominated by the neurons. Similar observations are made in the baboon data, where the glia do not pass the lognormality test, but the neurons do. In the cases of galago$_1$ and owl monkey both the neurons and glia pass the lognormality test (Fig. S2), which may, however, be partly due to the small number of density samples (*N* = 12 in both cases). Thus, the mouse and baboon data—with large samples sizes (*N* = 42 and *N* = 142, respectively)—suggest that it is the neuron densities that follow a lognormal distribution but not necessarily the glia densities.

Finally, we perform a control test on the different staining types—Nissl and NeuN—using the macaque$_1$ data. The staining methods differ in their treatment of glia: NeuN tends to label neuronal cell bodies only while Nissl indiscriminately labels both neurons and glia (Yurt et al. 2018). We show that regardless of staining type the cell densities pass the lognormality test (Fig. S3; SW test on $\ln (\rho )$ with $P>0.05$), suggesting that counting some glia in the cell densities does not confound our analysis of the macaque$_1$ data.

Taken together, the normality test, the quantile plots, the pairwise tests, the cell-type comparison, and the staining method comparison provide compelling evidence that the logarithmized neuron densities are normally distributed across cytoarchitectonic areas. This also holds for uniformly sampled neuron densities (baboon) but not for a sampling that is neither uniform nor based on a cytoarchitectonic parcellation (macaque$_2$, galago$_2$). Thus, the neuron densities are consistent with a lognormal distribution across the different cortical areas, as long as sampling is uniform.

**Fig. 2:**
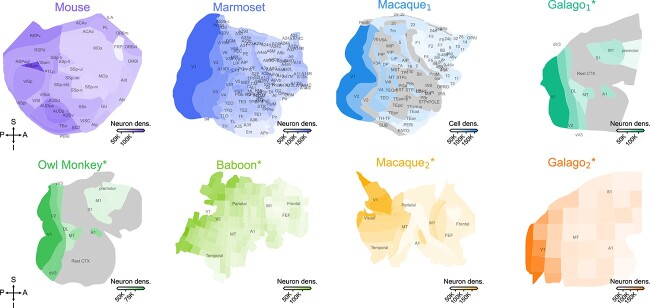
Neuron and cell densities $\rho $ over flattened cortical maps. Neuron and cell densities are color-coded, with darker colors indicating higher densities. Cortical maps not to scale due to the differences in brain size. All cortical flat maps are aligned in the same coordinate directions, showing a clear posterior–anterior gradient in all species. P: posterior, A: anterior, S: superior, I: inferior.

The observation of a lognormal distribution across cortical areas raises the question whether there is a spatial pattern of densities across cortex consistent for all species. To address this question, we visualized the neuron or cell density over flattened representations of the cortex for all data sets having a flat map (8 out of the 9 included in this study; Fig. 2). Consistent with previous reports (Collins et al. 2010; Erö et al. 2018; Atapour et al. 2019; Beul and Hilgetag 2019), we observe a clear posterior-to-anterior density gradient in all the species shown in this study. Visual areas display the highest densities, whereas motor and frontal areas display the lowest density.

### Lognormal distribution of neurons within marmoset cortical areas

**Fig. 3:**
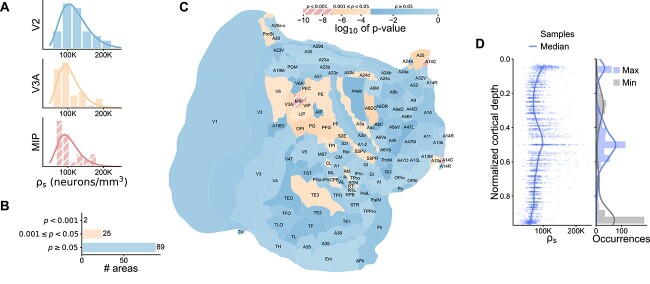
Neuron densities $\rho _s$ follow a lognormal distribution within most areas of marmoset cortex. (A) Sample histograms of $\rho _s$ and fitted lognormal distributions for 3 areas representing different degrees of lognormality. (B) Number of areas with *p*-values in the given significance ranges. (C) Log$_{10}$ of *p*-value of SW normality test of $\ln (\rho _s)$ on a flattened representation of the marmoset cortex (Atapour et al. 2019). (D) Scatter plot of sample densities against cortical depth and occurrence of highest and lowest density across normalized cortical depth (0 = L1/L2 boundary, and 1 = white matter boundary).

To investigate whether the lognormal distribution holds within cortical areas, we leverage detailed estimates of neuron density in marmoset (Atapour et al. 2019). Neurons were counted within $150\times 150\: {\mu{m}}$ counting frames for 4 strips per cortical area, all originating from the same subject. The within-area distributions of the sampled neuron densities $\rho _s$ across the counting frames in 3 representative areas (MIP, V2, and V3; Fig. 3A) again suggest a lognormal distribution. As before, we check for lognormality by testing $\ln (\rho _s)$ for normality with the SW-test (for full test results see Table S2). At significance level $\alpha = 0.05$, the normality hypothesis is not rejected for 89 out of 116 areas; whereas at $\alpha = 0.001$, this is the case for 114 out of 116 areas (Fig. 3B and C). Thus, regardless of the precise significance threshold, the lognormality hypothesis cannot be rejected within most cortical areas in the marmoset cortex. The contribution to the neuron density distributions differs across cortical depth: the highest densities tend to occur in Layer 2 or around the center of the gray matter depth, whereas the lowest values appear either in the upper layers or near the white matter boundary (Fig. 3D). Taken together, the findings from Figs. 1 and 3 show that the lognormal distribution of neuron densities can be found at different scales, both within and across the cortical areas.

### Statistical model comparison

**Fig. 4:**
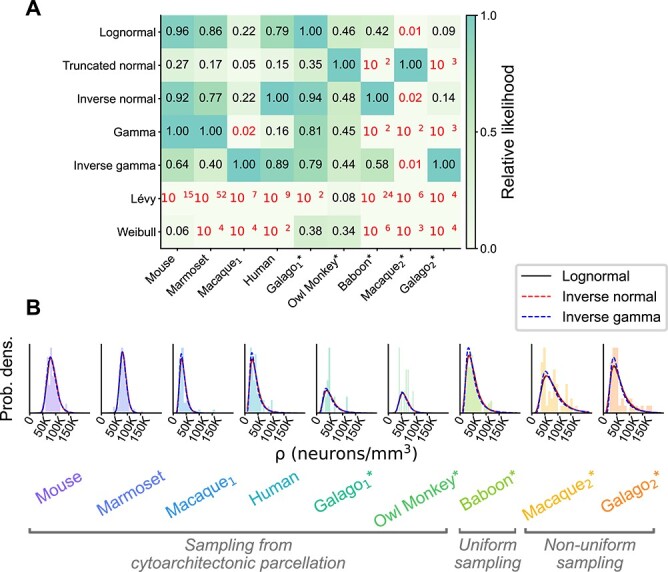
Statistical model comparison across the entire cortex of different animals. (A) Relative likelihood for 7 compatible statistical models for all available area-level neuron density data sets; numerical values indicated for each model and animal. The red color indicates a relative likelihood $<0.05$ with respect to the model with the highest likelihood. (B) The 3 best statistical models (according to the relative likelihood) fitted to the neuron density histograms in each animal; the 3 models produce visually nearly indistinguishable fits.

To complement the statistical hypothesis tests on the logarithmic densities, we compared the lognormal model with 6 other statistical distributions based on the relative likelihood (see Section Statistical model comparison). We included statistical distributions with support in $\mathbb{R}^+$ since neuron densities cannot be negative: lognormal, truncated normal, inverse normal, gamma, inverse gamma, Lévy, and Weibull. Of these, the lognormal, inverse normal, and inverse gamma distributions stand out as the distributions with the highest relative likelihoods, both across the entire cortex and within cortical areas (Fig. 4A, Fig. S4A). A visual inspection of the fitted distributions reveals that the lognormal, inverse normal, and inverse gamma distributions produce virtually indistinguishable probability densities (Fig. 4B, Fig. S4C); indeed, the relative likelihoods of the 3 models are above $0.05$ in all cases. This suggests that the data could theoretically be distributed according to either the lognormal, inverse normal, or inverse gamma distribution. To narrow down the model comparison, we show below how the lognormal distribution could arise from the simple biophysical process of noisy cell division. In contrast, we are not aware of a simple mechanism that could give rise to inverse normal or inverse gamma distributions.

**Fig. 5:**
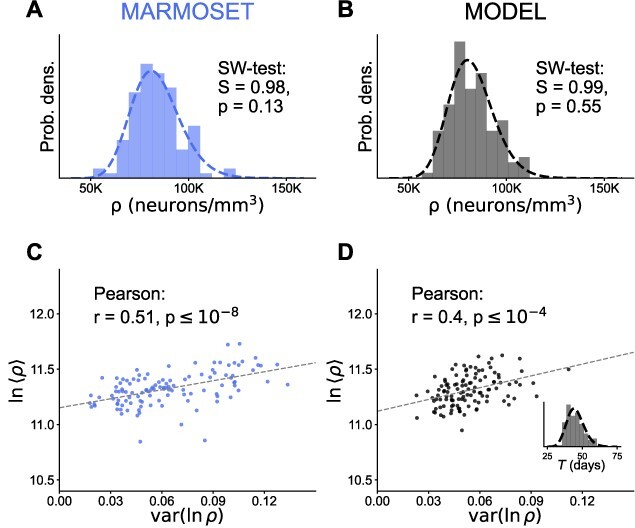
Neuron densities in the marmoset are compatible with a minimal model of neurogenesis with variability. (A, B) Distribution of neuron densities across areas for the marmoset (A) and the model (B). The SW test does not reject normality of $\ln (\rho )$ for both distributions. (C, D) The logarithmic mean $\ln (\langle \rho \rangle )$ and the variance of the logarithmic density $\text{var}(\ln (\rho ))$ for each area are significantly correlated with each other (one-sided Wald test with t-distribution) for the marmoset (C) and the model (D). The inset in (D) displays the inferred distribution (dashed line; see Equation (14)) of proliferation times alongside the sample used for the simulation (bars). For all parameters of the model and further details, see Model of neurogenesis with variability.

### A minimal model for the emergence of lognormally distributed neuron densities

The finding of lognormally distributed neuron densities raises the question how the intricate process of neurogenesis (Dehay and Kennedy 2007; Rakic 2009; Cadwell et al. 2019) culminates in this distribution within and across areas in several mammalian species.

On the within-area level, a minimal model shows that there is no need for a specific regulatory mechanism (see Model of neurogenesis with variability for further details): assuming that the proliferation of the neural progenitor cells is governed by a noisy rate $\lambda _{P}(t)+\xi (t)$, where $\lambda _{P}(t)$ denotes the mean rate and $\xi (t)$ is a zero-mean Gaussian white noise, the resulting density of progenitor cells is lognormally distributed.

The cells produced by the cell division of the neural progenitor cells are terminally differentiated neurons, which thus do not divide further. This renders the final neuron density a linear integral of the (changing) neural progenitor cell density. While this additive process formally does not preserve the lognormality of the neural progenitor density, the resulting neuron density distribution is statistically indistinguishable from a lognormal distribution with matched moments (SW test *P*  $=0.4$ with $N=2,000$ samples in Fig. S5F)—akin to the lognormal approximation for the sum of independent lognormal random variables (Fenton 1960; Marlow 1967). Put differently, the lognormally distributed density of neural progenitor cells leads to an approximately lognormally distributed density of neurons. Thus, the lognormal neuron density distribution within areas could be a hallmark of a progenitor cell proliferation process with variability.

On the across-area level, the proliferation times for each area become relevant, since they vary up to twofold (Rakic 2002). We therefore hypothesize that the distribution of proliferation times is the most important source of the variability across areas (variability due to area-specific proliferation rates (Lukaszewicz et al. 2005) is discussed below). Since the average neuron density within an area is determined by the proliferation time, the distribution of proliferation times specifies the distribution of area-averaged neuron densities across areas. This relation can be inverted to determine the distribution of proliferation times from the lognormal distribution of (within-area) average neuron densities (see Model of neurogenesis with variability). A specific prediction of this model is that the logarithmic mean $\ln (\langle \rho \rangle )$ and variance of $\ln (\rho )$ are related through the proliferation time—both mean and variability increase with proliferation time in approximate proportion to each other (see Equations (11) and (13), respectively). Indeed, we observe a linear correlation in the marmoset data (Pearson $r=0.51, \ P\leq 10^{-8}$, Fig. 5C) as well as in the data produced by the model (Pearson $r=0.4, \ P\leq 10^{-4}$, Fig. 5D).

## Discussion

In this work, we show that neuron densities are compatible with a lognormal distribution across cortical areas in multiple mammalian cortices and within most cortical areas of the marmoset, uncovering a ubiquitous organizational principle of cerebral cortex. The distributions of neuron and cell densities in general depend on the underlying spatial sampling: We found that neuron densities follow a lognormal distribution within cytoarchitectonically defined areas, across such areas, and when averaged within small parcels uniformly sampled across cortex, but not when sampled in a highly non-uniform manner not following cytoarchitectonic boundaries.

Furthermore, we show that none of a sizeable list of statistical models outperform the lognormal distribution. Our results are in agreement with the observation that surprisingly many characteristics of the brain follow lognormal distributions (Buzsáki and Mizuseki 2014). Moreover, this analysis highlights the importance of characterizing the statistical distributions of brain data because simple summary statistics—such as the mean or standard deviation—lack nuance and are not necessarily a good representation of the underlying data.

These findings are based on 9 publicly available data sets for 7 different species. While the majority of these have sample sizes in the range $36$–$114$, for completeness we also included 2 data sets consisting of $10$ samples each. As the latter sample size does not lead to a powerful test of lognormality, it would be desirable to repeat the analysis with more extensive data once available. In addition, data from multiple subjects of the same species would allow testing the consistency of the lognormal distribution across individuals.

Finally, we propose a minimal model that accounts for the emerging lognormal distributions based on a noisy cell division process of the neural progenitor cells and their specification to neurons. In principle, a multiplicative process with Gaussian noise leads to lognormal distributions at any scale; the additive specification from progenitors to neurons approximately preserves the lognormality. However, this noisy process alone cannot explain the coexistence of lognormal distributions of $\rho $ at different scales (within and across areas). When the distributed proliferation times are considered, the model can account for both within-area and across-area distributions.

The model explains the observed lognormality based on a minimal set of assumptions; hence, we did not include further mechanisms like cell death (apoptosis), migration, volumetric growth, the generation of other postmitotic cells, or area-specific proliferation rates. However, none of these additional mechanisms affect the main conclusions. Apoptosis is a widespread phenomenon (Oppenheim 1991; Inglis-Broadgate et al. 2005; Kalinichenko and Matveeva 2008) during neurogenesis and it can be modeled as a multiplicative process affecting the neuron density alone. Thus, the neuron density still depends linearly on the progenitor cell density and approximately maintains lognormality. While apoptosis could add to the inter-area variability, our model shows that it is not necessary for the distribution of variability per se—in agreement with Oppenheim et al. (1989) who found that the final pattern of spatial variability in spinal motoneuron density was already present before the onset of cell death and migration. Following the radial unit hypothesis (Rakic 2009), we focus on radial migration of progenitor cells, ignoring tangential migration. In our model, this assumption can be relaxed as long as there is no net increase or decrease in progenitor cell density due to tangential migration. Furthermore, we modeled the cell density in a fixed final target volume; thus, the effects of volumetric growth do not affect the model. The generation of other postmitotic cell types would reduce the effective proliferation rate of progenitor cells; this only leads to quantitative but not qualitative changes in the resulting distributions. Finally, it has been shown that cortical areas can have different proliferation rates (Polleux et al. 1997; Lukaszewicz et al. 2005; Dehay and Kennedy 2007). If the proliferation rates are constant in time and lognormally distributed across areas, this additional variability would broaden the neuron density distribution but preserve the lognormal shape. However, in contrast to distributed proliferation times, it would not lead to the correlation between mean density and variability seen in the data (Fig. 5C), because the proliferation rate affects the mean density but not the variance of the logarithmic progenitor density (see Eq. (7)). Furthermore, to the best of our knowledge, a difference in proliferation rate has only been shown for areas V1 and V2 (Lukaszewicz et al. 2005; Dehay and Kennedy 2007)—thus, we speculate that this is an important reason for the drastically higher neuron density in V1.

In contrast to the neuron densities, we observed that the densities of most glia types are not compatible with a lognormal distribution (Fig. S2). Across brain areas, our model requires distributed proliferation times to explain the emergence of the lognormal distribution of neurons. However, it is established that gliogenesis occurs after neurogenesis in both rodents and humans (Miller and Gauthier 2007; Semple et al. 2013), and glia are likely to have proliferation times distinct from those of neurons. Thus, given different glia proliferation times, our model does not necessarily predict a lognormal distribution across cortical areas, in agreement with the statistical tests from experimental data (Fig. S2). The principles governing the within-area distributions should, however, be very similar for both glia and neurons; thus, within cortical areas, our model predicts lognormal distributions of glial densities. Unfortunately, we are not aware of any experimental quantitative data on which this prediction could be tested.

Given the fact that cortical space is limited, one may expect a negative correlation between neuron density and soma size. We investigated this for the human data set, the only data set providing soma sizes. Indeed, there is a significant correlation (Pearson $r=0.67$, $P\le 10^{-30}$, Fig. S6) between inverse soma sizes and cell density in the human data set. Here, soma sizes were measured in terms of mean surface areas from the Nissl-stained slices by approximating the soma shapes as ellipses ($\pi /4$ times height times width). Since the cell densities were obtained linearly from the 2D densities, the total surface area taken up by the cells is proportional to their density times their mean surface area. Despite the positive correlation, this product is far from constant (Fig. S6), and hence also the area taken up by the extracellular space, neurites, and other structures such as blood vessels varies across areas and layers. To disentangle how these various factors are related to neuron density, joint measurements of the different components would be needed.

In principle, cortex-wide organizational structures might be by-products of development or evolution that serve no computational function (Otopalik et al. 2017)—but the fact that we observe the same organizational principle for several species and across most cortical areas suggests that the lognormal distribution serves some purpose. Heterogeneous neuron densities could assist computation through their association with heterogeneity in other properties such as connectivity and neuronal time constants (Rall 1969; Hilgetag et al. 2019); indeed, such heterogeneity is known to be a valuable asset for neural computation (Duarte and Morrison 2019; Perez-Nieves et al. 2021). Alternatively, localized concentration of neurons in certain areas and regions could also serve a metabolic purpose since centralization might support efficient transport of metabolites across neurons and astrocytes (Bélanger et al. 2011; Magistretti and Allaman 2015). Energy efficiency is particularly relevant since a large portion of the brain’s energy consumption is used to support the communication between neurons (Attwell and Laughlin 2001; Laughlin and Sejnowski 2003). Also from the perspective of cortical hierarchies it makes sense to have few areas with high neuron densities and many areas with lower neuron densities: Low-density areas contain neurons with large dendritic trees (Elston and Rosa 1998) receiving convergent inputs from many neurons in high-density areas lower in the hierarchy. The neurons with extensive dendritic trees in higher areas are involved in different, area-specific abstractions of the low-level sensory information (Kumar et al. 2007; Brincat et al. 2018). All in all, there is probably not a single factor that leads to lognormal neuron densities in the cortex; further research will be needed to refine our findings and uncover the functional implications.

## Supplementary Material

supplementary_materials_bhad160

suppl_data_bhad160

## Data and materials availability

This work produced no new data and instead relied on a corpus of neuron density data available from the literature, which we gratefully acknowledge; see Section Cell density data for a detailed description. The code used to analyze the data and create all figures is available in an open repository (DOI: 10.5281/zenodo.7871536).

## References

[ref1] Atapour N, Majka P, Wolkowicz IH, Malamanova D, Worthy KH, Rosa MG. Neuronal distribution across the cerebral cortex of the marmoset monkey (*Callithrix jacchus*). Cereb Cortex. 2019:29(9):3836–3863. 30357325 10.1093/cercor/bhy263

[ref2] Attwell D, Laughlin SB. An energy budget for signaling in the grey matter of the brain. J Cereb Blood Flow Metab. 2001:21(10):1133–1145. 11598490 10.1097/00004647-200110000-00001

[ref3] Beul SF, Hilgetag CC. Neuron density fundamentally relates to architecture and connectivity of the primate cerebral cortex. NeuroImage. 2019:189:777–792. 30677500 10.1016/j.neuroimage.2019.01.010

[ref4] Bélanger M, Allaman I, Magistretti P. Brain energy metabolism: focus on astrocyte-neuron metabolic cooperation. Cell Metab. 2011:14(6):724–738. 22152301 10.1016/j.cmet.2011.08.016

[ref5] Braitenberg V, Schüz A. Anatomy of the cortex: statistics and geometry. Berlin, Heidelberg, New York: Springer-Verlag; 1991.

[ref6] Braumann CA . Itô versus stratonovich calculus in random population growth. Math Biosci. 2007:206(1):81–107. 16214183 10.1016/j.mbs.2004.09.002

[ref7] Brincat SL, Siegel M, von Nicolai C, Miller EK. Gradual progression from sensory to task-related processing in cerebral cortex. Proc Natl Acad Sci. 2018:115(30):E7202–E7211. 10.1073/pnas.1717075115 PMC6064981 29991597

[ref8] Brodmann K . Vergleichende Lokalisationslehre der Großhirnrinde in ihren Prinzipien dargestellt auf Grund des Zellenbaues. Leipzig: Johann Ambrosius Barth; 1909.

[ref9] Burnham KP, Anderson DR, editors. Model selection and multimodel inference. New York: Springer; 2004.

[ref10] Buzsáki G, Mizuseki K. The log-dynamic brain: how skewed distributions affect network operations. Nat Rev Neurosci. 2014:15(4):264–278. 24569488 10.1038/nrn3687 PMC4051294

[ref11] Cadwell CR, Bhaduri A, Mostajo-Radji MA, Keefe MG, Nowakowski TJ. Development and arealization of the cerebral cortex. Neuron. 2019:103(6):980–1004. 31557462 10.1016/j.neuron.2019.07.009 PMC9245854

[ref12] Charvet CJ, Cahalane DJ, Finlay BL. Systematic, cross-cortex variation in neuron numbers in rodents and primates. Cereb Cortex. 2015:25(1):147–160. 23960207 10.1093/cercor/bht214 PMC4259279

[ref13] Collins CE, Airey DC, Young NA, Leitch DB, Kaas JH. Neuron densities vary across and within cortical areas in primates. Proc Natl Acad Sci. 2010:107(36):15927–15932. 20798050 10.1073/pnas.1010356107 PMC2936588

[ref14] Collins CE, Turner EC, Sawyer EK, Reed JL, Young NA, Flaherty DK, Kaas JH. Cortical cell and neuron density estimates in one chimpanzee hemisphere. Proc Natl Acad Sci. 2016:113(3):740–745. 26729880 10.1073/pnas.1524208113 PMC4725503

[ref15] Dehay C, Kennedy H. Cell-cycle control and cortical development. Nat Rev Neurosci. 2007:8(6):438–450. 17514197 10.1038/nrn2097

[ref16] Dombrowski S, Hilgetag CC, Barbas H. Quantitative architecture distinguishes prefrontal cortical Systems in the Rhesus Monkey. Cereb Cortex. 2001:11(10):975–988. 11549620 10.1093/cercor/11.10.975

[ref17] Dong HW . The Allen reference atlas: a digital color brain atlas of the C57Bl/6J male mouse. New York: John Wiley & Sons inc.; 2008.

[ref18] Dorkenwald S, Turner NL, Macrina T, Lee K, Lu R, Wu J, Bodor AL, Bleckert AA, Brittain D, Kemnitz N, et al. Binary and analog variation of synapses between cortical pyramidal neurons. Elife. 2022:11:e76120. 36382887 10.7554/eLife.76120 PMC9704804

[ref19] Duarte R, Morrison A. Leveraging heterogeneity for neural computation with fading memory in layer 2/3 cortical microcircuits. PLoS Comput Biol. 2019:15(4):e1006781. 31022182 10.1371/journal.pcbi.1006781 PMC6504118

[ref20] Elston GN, Rosa M. Morphological variation of layer III pyramidal neurones in the occipitotemporal pathway of the macaque monkey visual cortex. Cereb Cortex. 1998:8(3):278–294. 9617923 10.1093/cercor/8.3.278

[ref21] Erö C, Gewaltig M-O, Keller D, Markram H.A cell atlas for the mouse brain. Front Neuroinformatics. 2018:12:84. 10.3389/fninf.2018.00084 PMC6280067 30546301

[ref22] Ercsey-Ravasz M, Markov NT, Lamy C, Van Essen DC, Knoblauch K, Toroczkai Z, Kennedy H. A predictive network model of cerebral cortical connectivity based on a distance rule. Neuron. 2013:80(1):184–197. 24094111 10.1016/j.neuron.2013.07.036 PMC3954498

[ref23] Fenton LF . The sum of log-normal probability distributions in scatter transmission systems. IRE Trans Commun Syst. 1960:8(1):57–67.

[ref24] Gămănuţ R, Kennedy H, Toroczkai Z, Ercsey-Ravasz M, Van Essen DC, Knoblauch K, Burkhalter A. The mouse cortical connectome, characterized by an ultra-dense cortical graph, maintains specificity by distinct connectivity profiles. Neuron. 2018:97(3):698–715. 29420935 10.1016/j.neuron.2017.12.037 PMC5958229

[ref25] Herculano-Houzel S, Watson C, Paxinos G. Distribution of neurons in functional areas of the mouse cerebral cortex reveals quantitatively different cortical zones. Front Neuroanat. 2013:7(35). 10.3389/fnana.2013.00035 PMC3800983 24155697

[ref26] Hilgetag CC, Medalla M, Beul SF, Barbas H. The primate connectome in context: principles of connections of the cortical visual system. NeuroImage. 2016:134:685–702. 27083526 10.1016/j.neuroimage.2016.04.017 PMC5135480

[ref27] Hilgetag CC, Beul SF, van Albada SJ, Goulas A. An architectonic type principle integrates macroscopic cortico-cortical connections with intrinsic cortical circuits of the primate brain. Network Neurosci. 2019:3(4):905–923. 10.1162/netn_a_00100 PMC6777964 31637331

[ref28] Inglis-Broadgate SL, Thomson RE, Pellicano F, Tartaglia MA, Pontikis CC, Cooper JD, Iwata T. FGFR3 regulates brain size by controlling progenitor cell proliferation and apoptosis during embryonic development. Dev Biol. 2005:279(1):73–85. 15708559 10.1016/j.ydbio.2004.11.035

[ref29] Jardim-Messeder D, Lambert K, Noctor S, Pestana FM, de Castro Leal ME, Bertelsen MF, Alagaili AN, Mohammad OB, Manger PR, Herculano-Houzel S. Dogs have the most neurons, though not the largest brain: trade-off between body mass and number of neurons in the cerebral cortex of large Carnivoran species. Front Neuroanat. 2017:11:118. 29311850 10.3389/fnana.2017.00118 PMC5733047

[ref30] Kalinichenko SG, Matveeva NY. Morphological characteristics of apoptosis and its significance in neurogenesis. Neurosci Behav Physiol. 2008:38(4):333–344. 18401722 10.1007/s11055-008-0046-7

[ref31] Kornack DR, Rakic P. Changes in cell-cycle kinetics during the development and evolution of primate neocortex. Proc Natl Acad Sci. 1998:95(3):1242–1246. 9448316 10.1073/pnas.95.3.1242 PMC18732

[ref32] Kumar S, KE Stephan, JD Warren, KJ Friston, and TD Griffiths. June 2007. Hierarchical processing of auditory objects in humans. PLoS Comput Biol, 3 (6):e100. 17542641 10.1371/journal.pcbi.0030100 PMC1885275

[ref33] Laughlin SB, Sejnowski TJ. Communication in neuronal networks. Science. 2003:301:1870–1874. 14512617 10.1126/science.1089662 PMC2930149

[ref34] Lein ES, Hawrylycz MJ, Ao N, Ayres M, Bensinger A, Bernard A, Boe AF, Boguski MS, Brockway KS, Byrnes EJ, et al. Genome-wide atlas of gene expression in the adult mouse brain. Nature. 2007:445(7124):168–176. 17151600 10.1038/nature05453

[ref35] Liewald D, Miller R, Logothetis N, Wagner H-J, Schüz A. Distribution of axon diameters in cortical white matter: an electron-microscopic study on three human brains and a macaque. Biol Cybern. 2014:108(5):541–557. 25142940 10.1007/s00422-014-0626-2 PMC4228120

[ref36] Loewenstein Y, Kuras A, Rumpel S. Multiplicative dynamics underlie the emergence of the log-normal distribution of spine sizes in the neocortex in vivo. J Neurosci. 2011:31(26):9481–9488. 21715613 10.1523/JNEUROSCI.6130-10.2011 PMC6623170

[ref37] Lukaszewicz A, Savatier P, Cortay V, Giroud P, Huissoud C, Berland M, Kennedy H, Dehay C. G1 phase regulation, area-specific cell cycle control, and cytoarchitectonics in the primate cortex. Neuron. 2005:47(3):353–364. 16055060 10.1016/j.neuron.2005.06.032 PMC1890568

[ref38] Magistretti P, Allaman I. A cellular perspective on brain energy metabolism and functional imaging. Neuron. 2015:86(4):883–901. 25996133 10.1016/j.neuron.2015.03.035

[ref39] Markov NT, Ercsey-Ravasz M, Ribeiro Gomes AR, Lamy C, Magrou L, Vezoli J, Misery P, Falchier A, Quilodran R, Gariel MA, et al. A weighted and directed interareal connectivity matrix for macaque cerebral cortex. Cereb Cortex. 2014:24(1):17–36. 23010748 10.1093/cercor/bhs270 PMC3862262

[ref40] Marlow N . A normal limit theorem for power sums of independent random variables. Bell Syst Tech J. 1967:46(9):2081–2089.

[ref41] Miller FD, Gauthier AS. Timing is everything: making neurons versus glia in the developing cortex. Neuron. 2007:54(3):357–369. 17481390 10.1016/j.neuron.2007.04.019

[ref42] Oppenheim RW . Cell death during development of the nervous system. Annu Rev Neurosci. 1991:14(1):453–501. 2031577 10.1146/annurev.ne.14.030191.002321

[ref43] Oppenheim RW, Cole T, Prevette D. Early regional variations in motoneuron numbers arise by differential proliferation in the chick embryo spinal cord. Dev Biol. 1989:133(2):468–474. 2731638 10.1016/0012-1606(89)90050-x

[ref44] Otopalik AG, Sutton AC, Banghart M, Marder E. When complex neuronal structures may not matter. Elife. 2017:6:e23508. 28165322 10.7554/eLife.23508 PMC5323043

[ref45] Paxinos G, Watson CRR, Petrides M, Rosa MG, Tokuno H. The marmoset brain in stereotaxic coordinates. London: Elsevier; 2011.

[ref46] Perez-Nieves N, Leung VCH, Dragotti PL, Goodman DFM. Neural heterogeneity promotes robust learning. Nat Commun. 2021:12(1):5791. 34608134 10.1038/s41467-021-26022-3 PMC8490404

[ref47] Picco N, García-Moreno F, Maini PK, Woolley TE, Molnár Z. Mathematical Modeling of cortical neurogenesis reveals that the founder population does not necessarily scale with neurogenic output. Cereb Cortex. 2018:28(7):2540–2550. 29688292 10.1093/cercor/bhy068 PMC5998983

[ref48] Polleux F, Dehay C, Moraillon B, Kennedy H. Regulation of neuroblast cell-cycle kinetics plays a crucial role in the generation of unique features of neocortical areas. J Neurosci. 1997:17(20):7763–7783. 9315898 10.1523/JNEUROSCI.17-20-07763.1997 PMC6793912

[ref49] Rakic P . Neurogenesis in adult primate neocortex: an evaluation of the evidence. Nat Rev Neurosci. 2002:3(1):65–71. 11823806 10.1038/nrn700

[ref50] Rakic P . Oct 2009. Evolution of the neocortex: a perspective from developmental biology. Nat Rev Neurosci, 10 (10):724–735. 19763105 10.1038/nrn2719 PMC2913577

[ref51] Rall W . Time constants and electrotonic length of membrane cylinders and neurons. Biophys J. 1969:9(12):1483–1508. 5352228 10.1016/S0006-3495(69)86467-2 PMC1367649

[ref52] Razali NM, Yap BW. Power comparisons of Shapiro-Wilk, Kolmogorov-Smirnov, Lilliefors and Anderson-darling tests. J Stat Model Analytics. 2011:2(I):21–33.

[ref53] Robinson PA, Gao X, Han Y. Relationships between lognormal distributions of neural properties, activity, criticality, and connectivity. Biol Cybern. 2021:115(2):121–130. 33825983 10.1007/s00422-021-00871-z

[ref54] Santuy A, Rodríguez J-R, DeFelipe J, Merchán-Pérez A. Study of the size and shape of synapses in the juvenile rat somatosensory cortex with 3D electron microscopy. eNeuro. 2018:5(1):e0377–17.2017. 10.1523/ENEURO.0377-17.2017 PMC5790755 29387782

[ref55] Schultz-Darken N, Braun KM, Emborg ME. Neurobehavioral development of common marmoset monkeys: marmoset neurobehavioral development. Dev Psychobiol. 2016:58(2):141–158. 26502294 10.1002/dev.21360 PMC4829073

[ref56] Semple BD, Blomgren K, Gimlin K, Ferriero DM, Noble-Haeusslein LJ. Brain development in rodents and humans: identifying benchmarks of maturation and vulnerability to injury across species. Prog Neurobiol. 2013:106-107:1–16. 23583307 10.1016/j.pneurobio.2013.04.001 PMC3737272

[ref57] Turner EC, Young NA, Reed JL, Collins CE, Flaherty DK, Gabi M, Kaas JH. Distributions of cells and neurons across the cortical sheet in old world macaques. Brain Behav Evol. 2016:88(1):1–13. 27547956 10.1159/000446762

[ref58] Van Kampen NG . Stochastic processes in physics and chemistry. 3rd ed. Amsterdam: Elsevier; 2007.

[ref59] van Albada SJ, Morales-Gregorio A, Dickscheid T, Goulas A, Bakker R, Bludau S, Palm G, Hilgetag C-C, Diesmann M. Bringing anatomical information into neuronal network models. In: Giugliano M, Negrello M, Linaro D, editors. Computational modelling of the brain: modelling approaches to cells, circuits and networks. Springer International Publishing; 2022. pp. 201–234 10.1007/978-3-030-89439-9_9 35471541

[ref60] von Bartheld CS, Bahney J, Herculano-Houzel S. The search for true numbers of neurons and glial cells in the human brain: a review of 150 years of cell counting: quantifying neurons and glia in human brain. J Comp Neurol. 2016:524(18):3865–3895. 27187682 10.1002/cne.24040 PMC5063692

[ref61] von Economo C, Koskinas GN, Triarhou LC. Atlas of cytoarchitectonics of the adult human cerebral cortex. Heidelberg: Karger; 2008.

[ref62] Wang SS-H, Shultz JR, Burish MJ, Harrison KH, Hof PR, Towns LC, Wagers MW, Wyatt KD. Functional trade-offs in white matter axonal scaling. J Neurosci. 2008:28(15):4047–4056. 18400904 10.1523/JNEUROSCI.5559-05.2008 PMC2779774

[ref63] Yurt KK, Kivrak EG, Altun G, Mohamed H, Ali F, Gasmalla HE, Kaplan S. A brief update on physical and optical disector applications and sectioning-staining methods in neuroscience. J Chem Neuroanat. 2018:93:16–29. 29496551 10.1016/j.jchemneu.2018.02.009

