# Supplementary material for: Ubiquitous lognormal distribution of neuron densities in mammalian cerebral cortex
Source: Cereb Cortex. 2023 Jul 6;33(16):9439–49. doi: 10.1093/cercor/bhad160 (PMC10438924; doi:10.1093/cercor/bhad160)
Supplement: supplementary_materials_bhad160 [file supplementary_materials_bhad160.pdf]

Supplementary materials: Ubiquitous lognormal distribution of neuron densities in mammalian cerebral cortex

Aitor Morales-Gregorio[1,2*†], Alexander van Meegen[1,2†], Sacha J. van Albada[1,2]

[1]Institute of Neuroscience and Medicine (INM-6) and Institute for Advanced Simulation (IAS-6) and JARA-Institut Brain Structure-Function Relationships (INM-10), Jülich Research Centre, Jülich, Germany. [2]Institute of Zoology, University of Cologne, Cologne, Germany

[*]Corresponding author: a.morales-gregorio@fz-juelich.com
[†]These authors contributed equally to this work

Supplementary tables

Table S1: Cortical areas included in this study.

Species	Area abbreviations
Mouse	FRP, MOp, MOs, SSp-un, SSp-n, SSp-bfd, SSp-ll, SSp-m, SSp-ul, SSp-tr, SSs, GU, VISC, AUDd, AUDp, AUDpo, AUDv, VISal, VISam, VISl, VISp, VISpl, VISpm, ACAd, ACAv, PL, ILA, ORBl, ORBm, ORBvl, AId, AIp, AIv, RSPagl, RSPd, RSPv, PTLp, TEa, PERI, ECT
Marmoset	A10, A9, A46V, A46D, A8aD, A8b, A8aV, A47L, A47M, A45, A47O, ProM, A11, A13b, A13a, A13L, A13M, OPAl, OPro, Gu, A32, A32V, A14R, A14C, A25, A24a, A24b, A24c, A24d, A6DR, A6Vb, A6Va, A8C, A6M, A6DC, A4c, A4ab, PaIM, AI, PaIL, DI, GI, IPro, TPro, S2PR, A3a, S2PV, A3b, S2I, S2E, A1-2, AuRTL, AuRT, AuRPB, AuRTM, AuR, AuRM, AuAL, AuA1, AuCM, AuCPB, AuML, AuCL, TPPro, STR, TE1, TPO, ReI, TE2, PGa-IPa, TPt, TE3, TEO, Pir, APir, Ent, A36, A35, TF, TL, TH, TLO, TFO, A23c, A23a, A29d, A30, A23b, A29a-c, A23V, ProSt, PF, PE, PFG, A31, AIP, PG, PEC, VIP, LIP, PGM, V6A, OPt, MIP, MST, FST, V5, V4T, A19M, V3A, V4, V6, A19DI, V3, V2, V1
Macaque$_1$	2, 5, 9, 10, 11, 12, 13, 14, 23, 25, 32, 24a, 24c, 24d, 46d, 46v, 7A, 7B, 7m, 8B, 8l, 8m, 8r, 9-46d, 9-46v, DP, ENTO, F1, F2, F3, F4, F5, F6, F7, LIP, MT, OPAI, OPRO, PERI, STPi, TEad, TEav, TEO, TH-TF, V1, V2, V3A, V4
Human	FA, FB, FC, FCBm, FD, FDΔ, FDt, FE, FF, FG, FH, FJ, FK, FL, FM, FN, LA1, LA2, LC1, LC2, LC3, LD, LE1, LE2, IA, IB, OA, OB, OC, PA, PB1, PB2, PC, PD, PE, PF, PG, PH, HA, HB, HC, HD, HE, HF, TA, TB, TC, TD, TE, TF, TG
Galago$_1$ & Owl Monkey	V1, V2, dV3, vV3, S1, M1, A1, MT, premotor, DL

Table S2: Results of the Shapiro-Wilk test for normality of $\ln(\rho_s)$ in marmoset cortical areas. Values rounded to two significant digits.

**Area**	**S**	**p-value**	**Area**	**S**	**p-value**	**Area**	**S**	**p-value**
V1	0.97	0.39	AI	0.98	0.96	TH	0.97	0.66
A10	0.95	0.19	PaIL	0.95	0.33	TLO	0.96	0.18
A9	0.98	0.51	DI	0.96	0.45	TFO	0.97	0.26
A46V	0.98	0.56	GI	0.97	0.67	A23c	0.97	0.36
A46D	0.98	0.49	Ipro	0.97	0.66	A23a	0.99	0.98
A8aD	0.97	0.34	TPro	0.97	0.77	A29d	0.95	0.21
A8b	0.96	0.16	S2PR	0.92	0.006	A30	0.98	0.73
A8aV	0.96	0.17	A3a	0.95	0.04	A23b	0.97	0.45
A47L	0.96	0.052	S2PV	0.93	0.014	A29a-c	0.97	0.7
A47M	0.97	0.3	A3b	0.96	0.2	A23V	0.96	0.15
A45	0.96	0.18	S2I	0.97	0.33	ProSt	0.93	0.018
A47O	0.98	0.7	S2E	0.94	0.0046	PF	0.93	0.012
ProM	0.97	0.21	Area1-2	0.97	0.37	PE	0.94	0.03
A11	0.97	0.41	AuRTL	0.97	0.4	PFG	0.92	0.0046
A13b	0.96	0.58	AuRT	0.95	0.039	A31	0.97	0.31
A13a	0.91	0.048	AuRPB	0.98	0.89	AIP	0.96	0.063
A13L	0.97	0.45	AuRTM	0.97	0.73	PG	0.91	0.002
A13M	0.99	0.97	AuR	0.97	0.48	PEC	0.91	0.0032
OPAl	0.99	0.99	AuRM	0.9	0.017	VIP	0.92	0.0044
OPro	0.98	0.75	AuAL	0.94	0.12	LIP	0.95	0.042
GU	0.95	0.058	AuA1	0.98	0.48	PGM	0.98	0.78
A32	0.96	0.24	AuCM	0.97	0.33	V6A	0.95	0.068
A32V	0.96	0.51	AuCPB	0.93	0.037	OPt	0.91	0.0015
A14R	0.98	0.77	AuML	0.97	0.44	MIP	0.9	0.00091
A14C	0.79	5.5e-06	AuCL	0.94	0.045	MST	0.98	0.53
A25	0.89	0.022	TPPro	0.98	0.91	FST	0.95	0.1
A24a	0.96	0.35	STR	0.96	0.44	V5	0.98	0.68
A24b	0.97	0.41	TE1	0.96	0.17	V4T	0.95	0.082
A24c	0.97	0.54	TPO	0.97	0.31	A19M	0.98	0.8
A24d	0.92	0.017	ReI	0.95	0.4	V3A	0.91	0.006
A6DR	0.97	0.23	TE2	0.96	0.15	V4	0.98	0.61
A6Vb	0.97	0.32	PGa/IPa	0.97	0.45	V6	0.94	0.048
A6Va	0.98	0.56	TPt	0.94	0.033	A19DI	0.95	0.074
A8C	0.95	0.055	TE3	0.93	0.026	V3	0.97	0.53
A6M	0.99	0.98	TEO	0.95	0.087	V2	0.96	0.29
A6DC	0.91	0.002	A36	0.98	0.54	Ent	0.99	0.99
A4c	0.97	0.43	A35	0.97	0.31	APir	0.94	0.24
A4ab	0.96	0.076	TF	0.95	0.11	Pir	0.93	0.21
PaIM	0.93	0.2	TL	0.95	0.096			

Supplementary figures

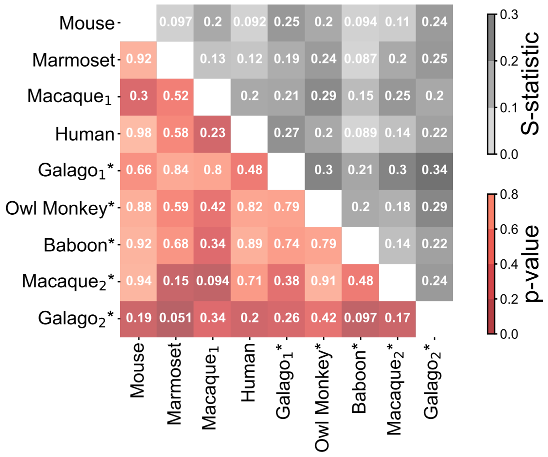

Figure S1: **Pairwise Kolmogorov-Smirnov two-sample two-sided tests.** P-values and S-statistics displayed below and above the diagonal, respectively. The z-scored log neuron density distributions of the four species are statistically indistinguishable at the 0.05 level.

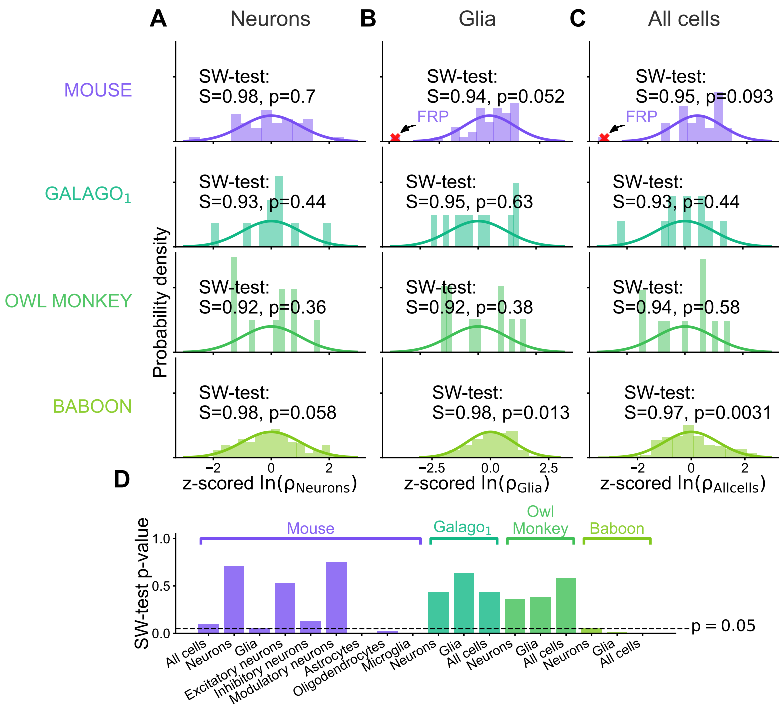

Figure S2: **Comparison of neuron and glia lognormality. A–C** Histogram of z-scored log density and result of Shapiro-Wilk test for neurons (**A**), glia (**B**), and all cells combined (**C**). **D** Barplot of p-values resulting from Shapiro-Wilk normality test for all cell types. Panel **A** is equivalent to the data shown in Figure 1.

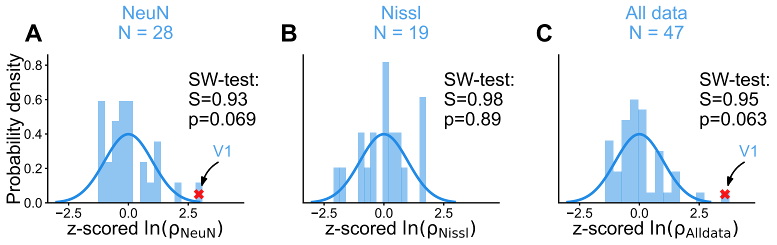

Figure S3: **Lognormality of cell densities from different staining types in macaque cortex based on the macaque$_1$ data set. A-C** Histogram of z-scored log density and result of Shapiro-Wilk test for NeuN staining only (**A**), Nissl staining only (**B**) and all measurements combined (**C**). The Nissl data were scaled down based on the linear relationship with the NeuN data (Beul and Hilgetag, 2019). Red crosses indicate outliers ($|$z-scored $\ln(\rho)| \geq 3$, which were excluded from the test. Panel **C** is equivalent to the data shown in Figure 1.

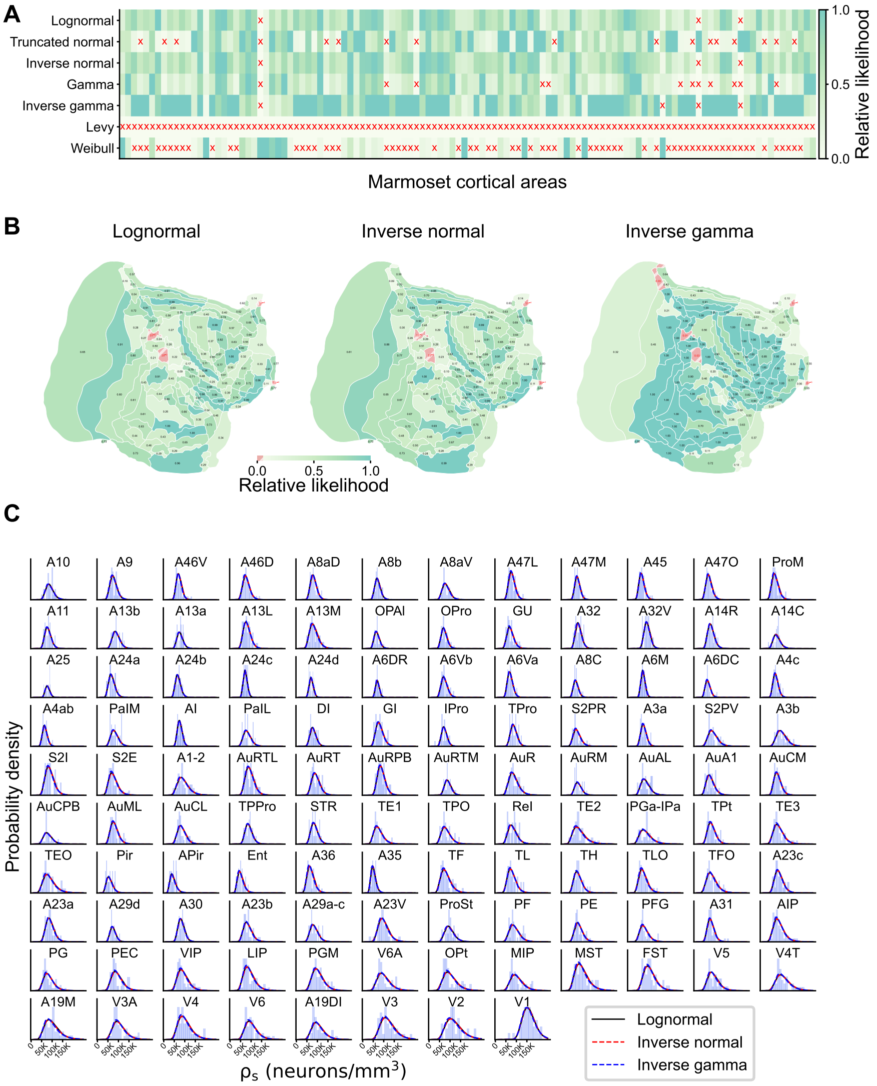

Figure S4: **Statistical model comparison within the marmoset cortical areas. A** Relative likelihood for seven compatible statistical models for all areas of the marmoset; a red cross (x) indicates a relative likelihood < 0.05 with respect to the model with the highest likelihood. **B** Spatial distribution of relative likelihood for the three best statistical models. **C** The three best statistical models fitted to the neuron density histograms in each area of marmoset cortex; the three models produce visually nearly indistinguishable fits.

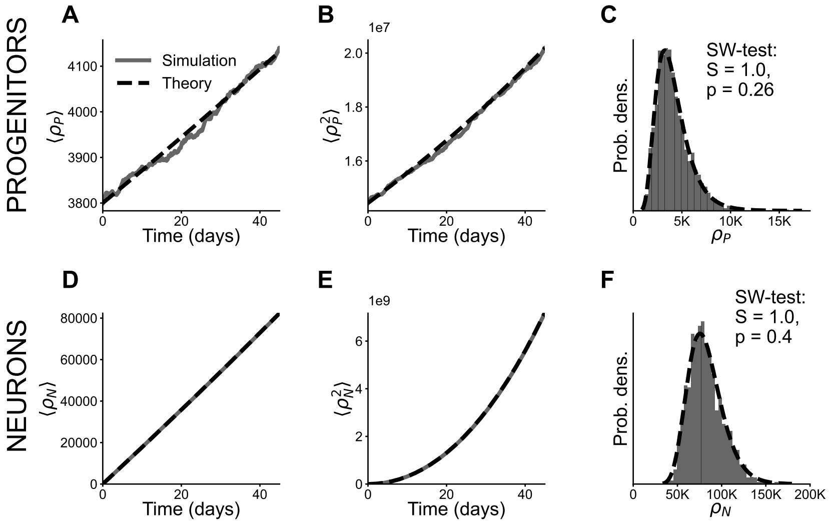

Figure S5: **Verification of neurogenesis model. A, B, D, E** Time evolution of the mean density of progenitors (A) and neurons (D) and the corresponding second moment for progenitors (B) and neurons (E). Theory based on Equations (8) and (10). **C, F** Resulting progenitor (C) and neuron (F) density distributions at the end of neurogenesis. Both distributions are compatible with a lognormal; for the neuron density this is not a formal result but rather an approximation based on Equation (11). Simulation parameters: time step $\Delta t = 0.05\,$days, total time $T = 45\,$days, $N_{\text{sample}} = 2000$ samples, initial progenitor density $\rho_0 = 3.8 \times 10^3$, average rate $\lambda = 2\log(2)/3\,$days, and noise intensity $\sigma = 0.061\,\text{days}^{-1/2}$.

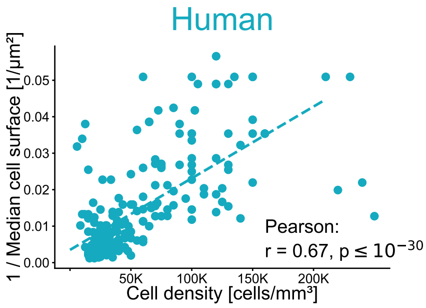

Figure S6: **Correlation between soma surface area and cell density in the human brain.** Each dot in the scatter plot represents a different area in human cortex. The strong positive correlation is confirmed by the one-sided Wald test with t-distribution.
